# Tailoring Polyamide66 Mechanical Performance: A Strategy for Condensed Phase Structure Optimization Through Hydrogen Bond Reorganization

**DOI:** 10.3390/molecules30040862

**Published:** 2025-02-13

**Authors:** Wen-Yan Wang, Pan He, Ting Peng, Shuai Zhang, Guang-Zhao Li, Min Nie, Rui Han

**Affiliations:** 1 School of Materials Science and Engineering, Key Laboratory of Materials and Surface Technology (Ministry of Education), Engineering Research Center of Intelligent Air-Ground Integration Vehicle and Control, Xihua University, Chengdu 610039, China; wwyandmmy@163.com (W.-Y.W.); penny_127@163.com (T.P.); brucezhang.scu@foxmail.com (S.Z.); guangzhao.li@hotmail.com (G.-Z.L.); 2 Sichuan Provincial Engineering Research Center of Functional Development and Application of High-Performance Special Textile Materials, Chengdu Textile College, Chengdu 611731, China; hepan-2009@163.com; 3 State Key Laboratory of Polymer Materials Engineering, Polymer Research Institute of Sichuan University, Chengdu 610065, China

**Keywords:** polyamide 66, TMB-5, condensed phase structure, mechanical performance

## Abstract

Polymers are widely used in various industries due to their unique properties, but their mechanical strength often falls short compared to other materials. This has spurred extensive research into enhancing their mechanical performance through condensed phase structure regulation. This study investigates the enhancement of mechanical properties in polyamide 66 (PA66) through the introduction of arylamide-based materials (TMB-5) during the melt-spinning process. TMB-5, possessing amide groups like PA66, can reorganize intermolecular hydrogen bonds within PA66, thereby facilitating molecular movement and reducing chain entanglement during fiber formation. Consequently, the synergistic effect of TMB-5 and the stretching field leads to enhanced crystallization and molecular and lamellae orientation in PA66 fibers without post-drawing, resulting in a significant increase in tensile strength and modulus. This work not only offers a novel strategy for adjusting polymer mechanical performance but also sheds light on the importance of molecular interactions in governing polymer properties.

## 1. Introduction

Polymers are extensively utilized in diverse industrial sectors owing to their unique combination of characteristics, such as lightweight nature, corrosion resistance, and tailored mechanical behavior [1,2,3,4,5]. In comparison to materials like metal and ceramic, polymers exhibit relatively weaker mechanical properties. Consequently, there has been considerable research interest in understanding and enhancing the mechanical attributes of polymers driven by the demand for materials with superior strength, stiffness, and durability [6,7,8]. Based on the current research, improving polymer performance can be summarized through three major pathways: multiphase structure design, main chain structure design, and control of the condensed phase structure. Multiphase structure design involves creating a material by combining different phases or materials, such as embedding fibers within the polymer matrix to improve its mechanical properties [9,10,11,12]. However, this method can introduce interface issues that could directly affect the performance of the final product. The interaction between the different phases is critical, and poor interfacial adhesion can lead to reduced mechanical properties [13,14,15]. From the perspective of chemical synthesis, the structure design of the polymer’s backbone is closely crosslinked with the properties [16,17,18]. It requires precise synthetic routes to ensure that the polymer chains have a consistent structure. The challenge here is to find a synthesis method that not only yields the desired structure but also has a high yield. If the synthesis path is not accurate, the polymer may not have the desired properties or may be produced in insufficient quantities. In contrast, the regulation of condensed phase structures mainly occurs during polymer processing. This involves using external fields (such as temperature, stress, or electric fields) to induce the formation of self-reinforcing condensed structures within the polymer, such as molecular chain orientation, high crystallinity, unique crystalline forms, and so on [19,20,21]. This method has unique advantages, including simplicity of process, strong design flexibility, and high reproducibility. Consequently, there is increasing attention on studying polymer performance from the perspective of condensed phase structures.

Among the condensed phase structures of polymers, adjusting the arrangement of molecular chains and crystallization behavior tends to significantly tailor their mechanical properties [22,23,24,25]. Research indicates that the application of stretching can alleviate polymer chain entanglement and align the polymer chains in the direction of the applied force, thereby increasing molecular orientation and even enhancing crystallization in certain polymers [26,27,28]. These mechanisms collectively enhance the load-bearing capacity of the polymer, thereby bolstering its overall strength. For instance, polylactide (PLA) films were subjected to intense extensional stress to tailor molecular orientation in the amorphous phase and induce crystallization, resulting in a significant enhancement of stiffness and strength in the absence of any filler introduction [28]. Additionally, advancements in processing techniques, such as melt-spinning, offer external fields for precisely controlling the condensed phase structures of polymers and further optimizing their mechanical properties [29,30,31,32]. Actually, the orientation of polymer chains needs to overcome intermolecular interactions and entanglements through the application of additional force. In other words, the degree of molecular chain orientation post-processing is intricately linked to intermolecular interaction. Surprisingly, there is a paucity of research focusing on the influence of regulating intermolecular interactions during polymer processing on the ultimate mechanical properties of the polymer, especially for polyamide 66 (PA66) containing a large number of hydrogen bonds. PA66 stands as a quintessential engineering plastic with robust intermolecular forces, owing to the presence of abundant amide groups along the molecular chain that facilitate hydrogen bond formation between PA66 molecular chains. While these robust intermolecular forces confer comprehensive performance on PA66, they simultaneously present a barrier to further achieving the regulation of condensed phase structures.

In polymer systems like PA66, regulating the intermolecular hydrogen bonds inevitably affects the molecular chain movement during the phase transition from a high-energy molten state to a low-energy solid state, as well as the final molecular chain conformation and crystal structure. An arylamide-based self-assembling β-nucleating agent (TMB-5), which contains amide groups similar to those in polyamide [33,34], was incorporated into the PA66 matrix. Materials with amide groups are known to form amide–amide hydrogen bonds with polyamide molecular chains [35,36]. Consequently, the incorporation of TMB-5 partially restricts the direct hydrogen bonding between PA66 molecular chains, resulting in an ‘internal plasticization effect’. This effect enhances the mobility and alignment of molecular chains within the PA66 melt during the melt-spinning process. As a result, the orientation of lamellae and the average length of stretched molecular chains in the PA66 fibers significantly improve. This leads to PA66 fibers with exceptional mechanical properties, achieving a remarkable tensile strength of 226.38 MPa without the need for post-drawing. This study sheds light on the intricate interplay between molecular interactions and mechanical properties of polymers, offering valuable insights into the development of high-performance polymers.

## 2. Experimental Section

### 2.1. Materials

Arylamide-based self-assembling β nucleating agent TMB-5 was provided by Shanxi Chemical Research Institute, Taiyuan, Shanxi, China. Its molecular structure is N,N′-dicyclohexyl-2,6-naphthalenedicarboxamide, as shown in Figure S1 [33,34]. Polyamide 66 (PA66), designated as A3401 and characterized by high viscosity for spinning, was sourced from BASF, Ludwigshafen, Germany.

### 2.2. The Preparation of TMB-5/PA66 Fiber

PA66 pellets were blended with TMB-5 powder at mass fractions of 0, 0.5, 1, 2, and 4 wt% (relative to the total mass) and subsequently co-mixed before being introduced into a twin-screw extruder. The extrusion process yielded TMB-5/PA66 particles containing varying mass fractions of TMB-5. The twin-screw extruder, model TSE-30A, was provided by Nanjing Ruiyafoster Polymer Equipment Co., Ltd., located in Nanjing, Jiangsu Province, China. The main screw speeds were set at 120 r/min and main feeding speed at 8 r/min. The extrusion temperatures ranged from 210 °C to 280 °C, progressing from the first to the final zones and the die, considering the water absorption tendencies of PA66. Prior to extrusion, the PA66 material, due to its high moisture absorption, underwent vacuum drying (The vacuum gauge reading is 0.08) at 80 °C for 8 h, maintaining a vacuum environment until the molding process to facilitate shaping. Similarly, the TMB-5 was vacuum-dried for 4 h before use. Some of these particles were directly compression-molded into sheets for comparison with the fiber properties.

**Figure 1:**
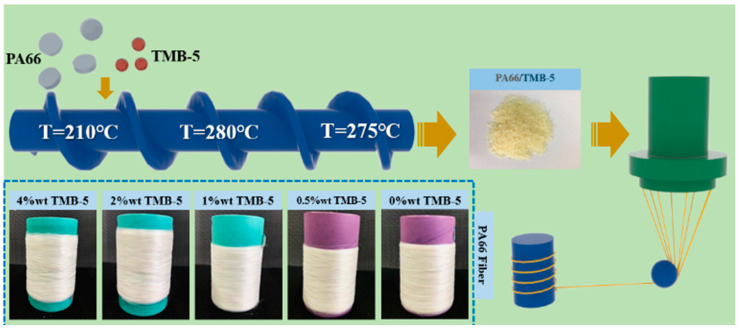
The preparation process of TMB-5/PA66 fibers.

Utilizing a single-screw extruder and metering pump, the previously prepared PA66 particles were melt-spun into monofilament fiber samples. The melt-spinning process operated at a temperature of 280 °C, with the screw pump rotating at a constant speed of 10 rpm. A nozzle with a diameter of 0.4 mm was employed for filament extrusion. The draw ratio (DR) was defined as DR = V1/V2, where V1 represented the winding speed and V2 the speed at the die exit; in this instance, DR = 64. The resulting filaments were respectively labeled as PT-X (where X denotes the mass fraction of TMB-5 in the TMB-5/PA66 fibers, e.g., PT-1). It should be noted that the PA66 fiber prepared in this study had the same draw ratio and did not undergo a post-drawing process. The schematic depiction of the preparation process is illustrated in Figure 1.

### 2.3. Characterization of the TMB-5/PA66 Fiber

The cross-sectional morphology and individual filament diameter of TMB-5/PA66 fibers were observed using a field emission scanning electron microscope (SEM, SU8010) operated at an acceleration voltage of 20 kV. Prior to this analysis, the fiber interfaces were subjected to gold sputter coating after fracturing in a liquid nitrogen environment. A polarizing microscopy (Leica DM2500P PLM) was used in conjunction with a hot stage (THMS600) to observe the migration and precipitation of TMB-5 at different temperatures ranging from 270 °C to 235 °C within TMB-5/PA66 blends. Dynamic mechanical analysis (DMA, DMA-Q800) in tensile mode was employed to evaluate the viscoelastic properties of TMB-5/PA66 fiber specimens. Testing was conducted at a frequency of 1 Hz over a temperature range of −100 °C to 120 °C, with a heating rate of 3 °C/min. The melting and crystallization behavior of TMB-5/PA66 fiber specimens were investigated using differential scanning calorimetry (DSC, DSC-25) under a nitrogen atmosphere. The temperature was increased from 40 °C to 290 °C at a rate of 10 °C/min.

Characterization of the crystal lattice structures of the prepared PA66/TMB fibers was carried out using 2D wide-angle X-ray diffraction (2D-WAXD) at the Shanghai Synchrotron Radiation Facility. X-ray wavelength was set at 0.124 nm, with a circular beam size of 1 mm in diameter. The distance between the sample and the CCD detector was 259 mm. Measurements were conducted at room temperature in transmission mode, and data were recorded using Pilatus1M CCD and analyzed using Xpolar analysis software version 4.5.14. Additionally, the stretch-induced chain segment length (S) and lamellae periodicity (L) of the fabricated PA66/TMB fibers were determined using 2D small-angle X-ray scattering (2D-SAXS) at the BL16B1 beamline of the Shanghai Synchrotron Radiation Facility. X-ray wavelength and beam size were maintained at 0.124 nm and 1 mm in diameter, respectively. The sample-to-CCD distance was 1948 mm, with measurements conducted at room temperature in transmission mode. Data were recorded using either Mar165 or Pilatus1M CCD and analyzed using Xpolar analysis software. The TMB-5/PA66 fibers were subjected to orientation measurements using the Donghua Kelly SCY-III Sonic Velocity Fiber Orientation Meter. The fibers were conditioned for 24 h at approximately 25 °C and 60% relative humidity, spanning a length of 80 cm with a gauge length of 40 cm, to determine the non-oriented sonic velocity Cu. Subsequently, the orientation factor was computed using the Formula (1) [32]:

(1) $$f_{s}=1-\frac{C_{U}^{2}}{C^{2}}$$

Here, $f_{s}$ represents the orientation factor and C denotes sonic velocity [32]. An electronic universal testing machine (CMT6104, SANS Test & Measurement Technology (Zhejiang) Co., Ltd., Jiaxing, China) was employed to test the tensile properties of PA66 sheets with a stretching rate of 40 mm/min. The single-fiber tensile strength testing of the fibers was performed at room temperature using a New Fiber XQ-2 Single Fiber Tensile Tester (Shanghai New Fiber Instrument Co., Ltd., Shanghai, China), employing a stretching rate of 40 mm/min and a clamping distance of 10 mm.

## 3. Results and Discussion

### 3.1. Morphological Analysis of TMB-5 in TMB-5/PA66 Fibers

**Figure 2:**
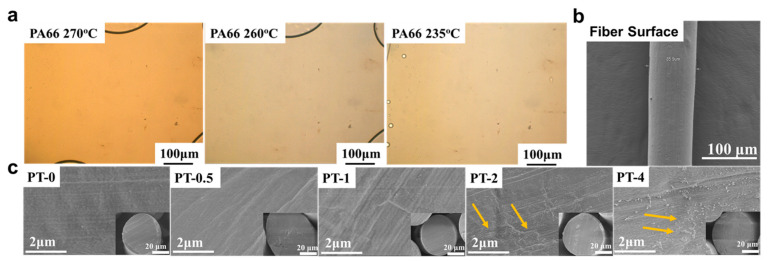
The POM images during the cooling process of melted PA66 fibers containing 4 wt% TMB-5 (**a**), the surface SEM image of a single PA66 fiber (**b**), the SEM image of the cross-section of TMB-5/PA66 fibers (**c**).

Figure 2a shows the POM images during the cooling process of melted PA66 fibers containing 4 wt% TMB-5. It is observed that upon heating to 270 °C, the TMB-5 dissolves completely within the PA66 melt, rendering the melt optically clear. Given the substantial presence of amide groups in PA66 molecular chains, they share similar chemical properties with TMB-5, readily forming stable hydrogen bonds that induce effective ‘solvation’ of TMB-5 molecules within PA66. Consequently, during the cooling phase, TMB-5 fails to self-assemble before PA66 crystallization, remaining dispersed in the form of small molecules within PA66.

Figure 2b presents SEM images illustrating the surface morphology of individual TMB-5/PA66 fibers, displaying a uniform contour without noticeable defects such as pores or protrusions. For further investigation of internal morphology in different TMB-5/PA66 fibers obtained from melt-spinning, the fractured cross-sections of the fibers were observed, as shown in Figure 2c. The micrographs reveal that fibers with lower TMB-5 concentrations, such as PT-0 and PT-0.5, exhibit smoother cross-sections devoid of significant phase separation or particles. Conversely, fibers with higher loaded TMB-5 concentrations, like PT-2 and PT-4, display evident punctate and aggregated structures on their cross-sections. Some researchers have previously confirmed through polarized light microscopy that certain nucleating agents with intermolecular forces can self-assemble into diverse topological structures during the polymer melt–cool process [37,38]. During the melt-spinning of PA66, the dissolved TMB-5 within PA66, at lower concentrations, is easily dispersed among PA66 molecular chains, forming stable hydrogen bonds with PA66 molecular chains during fiber cooling and solidification. This aspect restricts the likelihood of small TMB-5 molecules aggregating within the amorphous regions between crystalline domains due to PA66 crystallization. Additionally, it slows down the kinetics of TMB-5 molecule migration, aggregation, and self-assembly within PA66. However, at higher TMB-5 concentrations, the TMB-5 molecules are more prone to being squeezed into the interlamellar regions of PA66 during cooling and solidification, substantially increasing the probability of forming hydrogen bonds between their own molecules. This notably accelerates the kinetics of self-assembly and precipitation, showcasing diverse self-assembled morphologies with varying concentrations. These internal particles within PA66 fibers tend to become stress concentration points during fiber loading, potentially exerting adverse effects on the macroscopic performance of the fibers.

### 3.2. The Crystallization Behavior of TMB-5/PA66 Fibers

**Figure 3:**
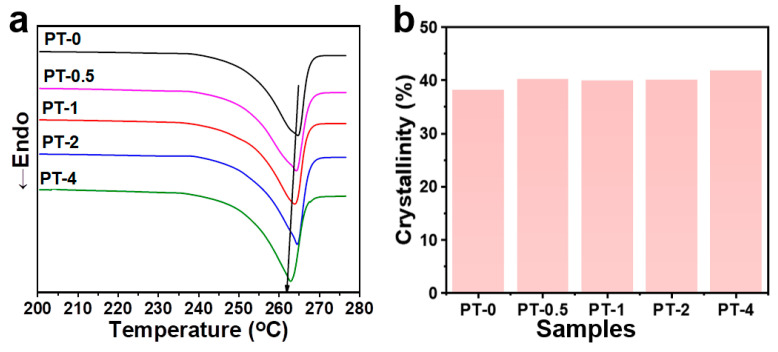
The DSC endothermic (Endo) curves (**a**) and the summarized crystallinity (**b**) of TMB-5/PA66 fibers.

Figure 3a presents the DSC test results of PA66 fibers with different masses of TMB-5 after melt-spinning. Contrasting with the dual melting peaks observed in PA66 sheets (Figure S2), the fibers’ melting curve exhibits only one melting peak, with a peak temperature around 263 °C (Figure 3a), indicating the presence of a single α2-type melting peak for the PA66 fiber [39]. This suggests that the flow-induced crystallization process occurred in PA66 fibers due to shear in the spinning device followed by rapid stretching, where the elongated molecular chains promoted the growth of PA66 crystals, leading to the formation of a thermodynamically more stable α2-type crystal structure. According to the related DSC results (Figure 3a and Table S1), with the increase in TMB-5, the fibers’ melting peak slightly shifts to lower temperatures, indicating a decrease in perfection of crystallites resulting from steric hindrance of TMB-5 to some extent. The fibers’ melting enthalpy increases with increasing TMB-5 content, and the crystallinity $X_{C}$ is calculated using Formula (2) [40]:

(2) $$X_{C}=∆H_{m}/(1-\varphi )∆H_{m}^{0}\times 100\%$$

Here, $∆H_{m}$ represents the sample’s melting enthalpy, $∆H_{m\;}^{0}$ is the standard melting enthalpy of PA66, and *φ* is the content of the TMB-5. The calculated crystallinity is summarized in Figure 3b, showing an increasing trend in fiber crystallinity (from 38% to 43%). This is attributed to the presence of TMB-5 molecules, which facilitate the movement and straightening of molecular chains in the PA66 melt during fiber formation, making it easier for crystallites and amorphous regions of PA66 fibers to move during thermal stretching. Therefore, a competitive relationship is observed in TMB-5/PA66 fibers, whereby the weakened non-isothermal crystallization ability due to the hindered formation of hydrogen bonds between PA66 molecular chains competes with the enhanced stretching-to-crystallization ability caused by chain mobility. Based on the current results, although these two effects induced by TMB-5 in the PA66 fiber formation process reduce the perfection of crystallites, they lead to a slight increase in overall crystallinity.

### 3.3. The Orientation of the Condensed Phase Structure in TMB-5/PA66 Fibers

**Figure 4:**
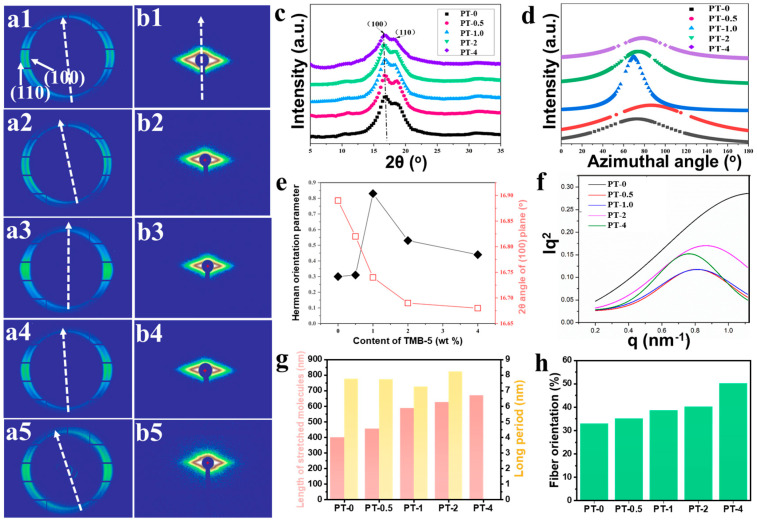
The 2D-WAXD (**a1**–**a5**), 2D-SAXS (**b1**–**b5**) test results, (**c**,**d**) show the results obtained from the 1D integration of 2θ angle and PA66 (100) crystal plane for the 2D images of (**a1**–**a5**), respectively. Herman’s orientation factor of PA66 fiber (**e**) and the relative q-value curves (**f**) obtained using 2D-SAXS (**b1**–**b5**). The length of stretched molecules and long period information inside PA66 fibers (**g**) and the fiber orientation factor measured using the sound velocity method (**h**).

Further exploration of the structural evolution at the crystallographic scale in PA66 fibers with varying TMB-5 content was conducted using 2D-WAXD, as depicted in Figure 4(a1–a5). The scattering rings corresponding to each sample in Figure 4(a1–a5) exhibit non-uniform distribution, with diffraction arcs appearing near the equator line, indicating a certain degree of oriented arrangement of PA66 lamellae within the fibers. It is important to note that the diffraction arcs in Figure 4(a1–a5) are not perfectly aligned along the horizontal line due to the flexibility of the fibers, making it challenging to ensure their vertical alignment to the ground during sample placement. To rectify this issue, the actual orientation of the fiber axes for each sample was indicated in the figure. Comparing the diffraction patterns in Figure 4(a1–a5), a noticeable trend is observed as the TMB-5 content increases, with the diffraction arcs showing a gradual concentration and subsequent dispersion. This suggests a variation in the orientation degree of PA66 lamellae along the fiber axis, initially increasing and then decreasing with rising TMB-5 content.

This conclusion was further validated through quantitative analysis. Figure 4c,d depict the one-dimensional integration of the 2θ angle and PA (100) crystal plane obtained from the two-dimensional images in Figure 4(a1–a5) respectively. The results reveal that the crystal structures in all PA66 fibers exhibit typical α-type crystals, featuring diffraction peaks of (100) and (110) crystal planes at around 16.7° and 18.2°, respectively. The peak intensities of the (100) and (110) crystal planes in the one-dimensional curves indicate weakened intensities for all fibers, suggesting a significant disruption in crystal size and integrity of PA66 fibers after stretching during the spinning process. Moreover, a reduction in the diffraction angle of the (100) crystal plane is observed with increasing TMB-5 content, shifting from 16.89° in the PT-0 sample to 16.68° in the PT-4 sample (Figure 4e). According to Bragg’s law, this reduction implies a proportional relationship between the interplanar spacing and the amount of added TMB-5, signifying that a higher TMB-5 content results in increased interplanar spacing [41]. This also indicates that the inclusion of TMB-5 exacerbates the crystal fracture of fibers under stretching effect during melting spun. Furthermore, utilizing the (100) crystal plane as an indicator, Herman’s orientation factor was employed for a quantitative comparison of the orientation degree of lamellae within PA66, and the calculation process are supplied in the Supporting Information [23,42,43]. As for the results, they are shown in Figure 4e. It is evident that the orientation degree of lamellae within the fiber first increases and then decreases with the rising TMB-5 content. In the PT-1 sample, the orientation factor reaches its maximum value of 0.83, marking a substantial increase of 177% compared to the pure PA66 fiber’s value of 0.30. This further substantiates that the unique molecular structure of TMB-5 is advantageous for enhancing the molecular motion orientation during the formation of PA66 fibers. However, the Herman’s orientation factor for PT-2 and PT-4 samples does not show further improvement. This can be attributed to the excessively high TMB-5 content leading to the presence of numerous lattice defects introduced by TMB-5 small molecules within the lamellae. During melting spun process, the lamellae are more prone to fracture, forming smaller crystalline blocks. As the fiber cross-section continuously converges, this results in a more pronounced deviation motion, adversely affecting their ordered arrangement.

2D-SAXS testing provides insights into the larger-scale condensed structures within PA66 fibers. As observed in Figure 4(b1–b5), the prepared PA66 fibers, regardless of the presence of TMB-5, exhibit non-circular and uniformly distributed scattering patterns of anisotropic structures. The scattering intensity in the meridional direction significantly diminishes, while the intensity in the equatorial direction noticeably increases. Considering the fibers are vertically oriented, this implies the existence of numerous condensed structures aligned along the fiber axis, likely formed during the continuous stretching process, representing an aggregation of stretched molecular chains. In this study, all orientation distributions exhibited good fits, and the resulting average lengths of stretched molecular chains are shown in Figure 4g and Table S2 (the fitting calculation process is in Supporting Information). Consistent with the earlier conclusions, the addition of TMB-5 reduces the intermolecular forces between large molecular chains, expanding the free volume for molecular chain movement and slip during the formation of PA66 fibers. This enhancing effect becomes more pronounced with an increasing amount of added TMB-5, leading to a gradual increase in the average length of stretched molecular chains from 400 nm to 671 nm in the final sample, as observed in the PT-4 sample.

Figure 4f is the relative q-value curves obtained from 2D-SAXS (Figure 4(b2–b5)) and these values are used in the relevant long-period calculation process appearing in Supporting Information. As depicted in Figure 4g and Table S2, with an increasing amount of TMB-5, the long period in PA66 fibers exhibits an initial decrease followed by an increase (PT-4 sample cannot be explicitly probed). This result once again indicates that TMB-5 plays two conflicting competitive roles in the crystallization process of PA66 fibers. On one hand, it promotes crystallization by facilitating the movement and stretching of molecular chains; on the other hand, it hinders crystallization by occupying positions within the lattice. Evidently, when the TMB-5 content is relatively low, the promotion of crystallization prevails, facilitating better preservation of the crystal structure under stretching effect from melting spun in the later stage of fiber formation. However, when the TMB-5 content is high, the inhibitory effect on crystallization dominates, leading to the destruction of the crystal structure during melting spun process. At a TMB-5 addition of 4 wt%, the long-period structure becomes significantly challenging to observe in the fiber samples.

As a validation, the sound velocity method was employed to measure the fiber orientation factor [32,44]. This method quantitatively assesses the average orientation factor of large molecular chains within the fiber by exploiting the difference in sound propagation velocities along the main chain direction and perpendicular direction. It reflects the statistical average of the angles between the main molecular chains and the fiber axis. A higher orientation factor obtained through the sound velocity method indicates a greater orientation of the large molecular chains along the fiber axis. Figure 4h and Table S3 illustrates the orientation factors of fibers measured using the sound velocity method. The data indicates an increase in the orientation of molecular chains within the fiber with the growing TMB-5 concentration. The orientation factor rises from 0.329 in the PT-0 sample to 0.502 in the PT-4 sample, further confirming that the TMB-5 effectively promotes molecular chain movement, crystallite fragmentation, and enhances the axial orientation of molecular chains in PA66 fibers during the formation process. However, it is essential to note that the overall orientation factor of large molecular chains in the fibers studied in this research is not exceptionally high. This is attributed to the fact that the PA66 fibers prepared in this study did not undergo post-drawing and thermal setting, resulting in a relatively limited presence of stretched molecular chains within the fibers. Additionally, the abundance of fractured crystallites contributes to a lower orientation factor measured through the sound velocity method, as the molecular chains continuously fold and unfold within the crystallites.

### 3.4. The Dynamic Mechanical Properties of TMB-5/PA66 Fibers

**Figure 5:**
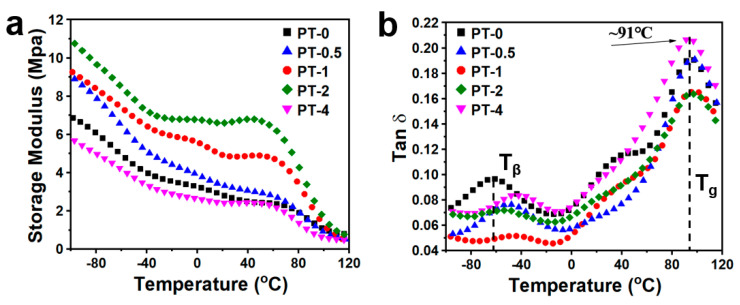
The DMA curves for TMB-5/PA66 fibers: (**a**) the storage modulus, (**b**) the loss factor.

This study investigated the dynamic mechanical properties of TMB-5/PA66 fibers. Figure 5a and Figure 5b respectively present the temperature-dependent curves of the storage modulus (E′) and the loss factor (tanδ) for TMB-5/PA66 fibers. From Figure 5a, it can be observed that, except for the PA66 fibers with a 4 wt% TMB-5, the storage modulus of fibers with other concentrations is higher than that of samples without the TMB-5. This indicates that during the fiber formation process, the uniform distribution of TMB-5 small molecules promotes the movement of PA66 molecular chains, thereby exerting a positive effect on the oriented arrangement and flow of molecular chains towards crystallization, which are consistent with DSC and 2D-WAXD results. Additionally, it is evident that within the temperature range of 25 °C to 50 °C, as the TMB-5 concentration gradually increases from 0%wt to 4%wt, the decrease in storage modulus with temperature becomes more moderate, with even slight increases observed in the 1%wt and 2%wt samples. This suggests that the enhanced crystallinity and orientation introduced by TMB-5 during the PA66 fiber formation process result in improved stiffness and deformation recovery capability of the fibers within the temperature range below the glass transition.

In Figure 5b, it can be observed that with an increase in the concentration of TMB-5, the secondary transition temperature (this temperature is usually labeled as $T_{\beta }$ [45]) of PA66 fibers shifts towards higher temperatures, enhancing the stability of PA66 fibers at low temperatures. Regarding the glass transition temperature ($T_{g}$), the fiber’s $T_{g}$ temperature reaches around 91 °C, with no significant correlation observed with TMB-5 content. When compared with the glass transition temperature of approximately 63 °C for TMB-5/PA66 sheets in Figure S3, the $T_{g}$ of PA66 fibers shows a significant improvement, mainly due to the more ordered arrangement of molecular chains and a significant reduction in molecular chain free volume induced by the stretching force field during fiber thermal forming. It is also noteworthy that in pure PA66 fibers, a distinct shoulder peak is observed within the temperature range of 25 °C to 50 °C, which gradually disappears with increasing TMB-5 content. This further underscores the important role of TMB-5 addition in enhancing the orientation and compact arrangement of molecular chains in the fibers, thereby to some extent inhibiting the thermal relaxation of certain smaller unit chain structures within PA66 fibers.

### 3.5. The Tensile Performance and Mechanism of TMB-5/PA66 Fibers

**Figure 6:**
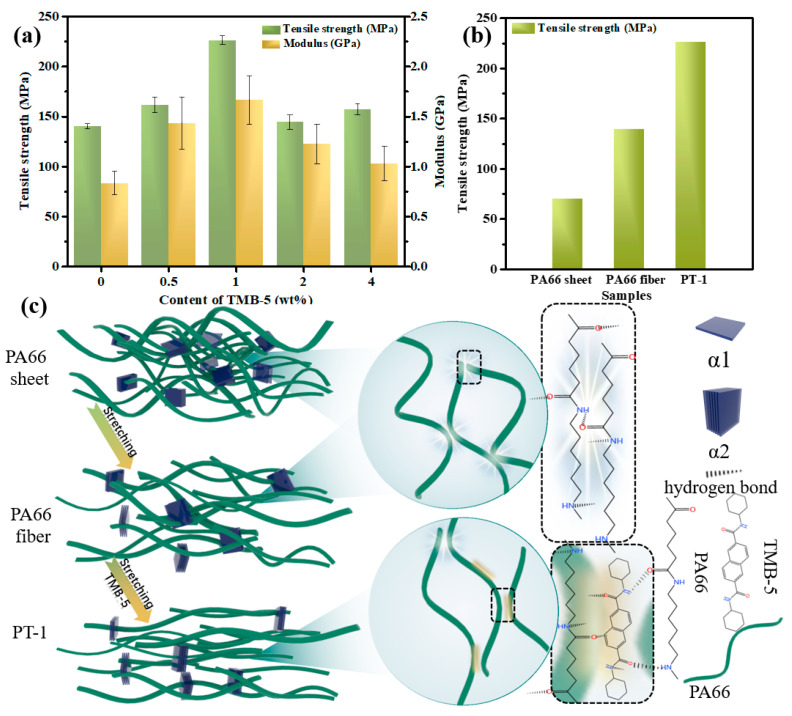
The mechanical properties of PA66 fibers (**a**), comparison of tensile strength between PA66 sheet, PA66 fiber, and PT-1 (**b**) and the schematic diagram illustrating PA66 microstructure evolution (**c**).

It can be clearly known that the condensed phase structure of PA66 fiber changed as a joint result of stretching and introduction of TMB-5 based on the previous analysis. Therefore, we conducted further evaluations on the tensile properties of PA66 materials with different TMB-5 concentrations, as depicted in Figure 6a and Figure S4. Obviously, the mechanical properties of PA66 fibers significantly surpass those of PA66 sheets. For instance, while the tensile strength of pure PA66 sheet is only 71 MPa, the pure PA66 fiber exhibits an outstanding tensile strength of 140.14 MPa. This observation directly underscores that stretching is an effective means to enhance the mechanical properties of the polymer. Interestingly, the introduction of TMB-5 has a negligible effect on the tensile performance of PA66 sheets without the influence of stretching (Figure S4). However, the tensile performance of fibers with increasing TMB-5 content demonstrates an initial increase followed by a decrease trend. Specifically, the tensile strength increases from 140.14 MPa for pure PA66 to 226.38 MPa for the PT-1 material. Similarly, the elastic modulus exhibits a similar changing trend. Overall, PA66 with an appropriate amount of added TMB-5 demonstrates a significant enhancement in tensile performance after melt-spinning, achieving optimal tensile strength and modulus increases of 61.5% and 107.1%, respectively, compared to pure PA66 fibers. This indicates a synergistic effect between the stretching field and TMB-5 on mechanical performance. However, exceeding a TMB-5 concentration of 2 wt% results in a significant decrease in fiber tensile performance. This decline can be attributed to the self-assembly of TMB-5, as evidenced in Figure 2c. These internal particles within the PA66 fibers serve as stress concentration points, ultimately compromising the mechanical properties of the fibers. Therefore, an optimal TMB-5 concentration exists for PA66 fibers, determined to be 1wt% in this study.

To thoroughly explain the mechanism behind PA66 reinforcement, the schematic diagram illustrating the PA66 condensed phase structure evolution is presented in Figure 6c. In the PA66 sheet, molecular chains exhibit a tendency to coil and form pronounced entanglements. Moreover, the PA66 sheet contains two distinct crystal forms, as revealed by DSC analysis: thin α1-type crystals and thicker α2-type crystals. Amide groups on PA66 molecular chains facilitate hydrogen bonding between intermolecular chains, resulting in robust interaction and conferring the PA66 sheet with a good tensile strength of 71.0 MPa (Figure 6b). In contrast, the micromorphology of PA66 fiber, prepared via melt-spinning, differs from that of the PA66 sheet. The stretching effect during the preparation process significantly contributes to the orientation of PA66 molecular chains and diminishes their entanglement, leading to a higher tensile strength of PA66 (140.1 MPa, Figure 6b). Moreover, the presence of single α2-type crystals provides further reflection of enhanced molecular chain orientation. Meanwhile, hydrogen bonds still exist in intermolecular chains of PA66. Upon introducing TMB-5 into PA66 fiber, these hydrogen bonds between intermolecular chains of PA66 are partially replaced by those formed between TMB-5 and PA66 molecular chains. This substitution further facilitates the movement and straightening of molecular chains in the PA66 melt during fiber formation, as proved in Section 3.3. Benefiting from the synergistic effect of TMB-5’s internal plasticization and stretching during melt-spinning, the orientation degree of lamellae and the average length of stretched molecular chains in PA66 fibers without post-drawing increase significantly, resulting in a remarkably high tensile strength of 226.4 MPa for PA66. It is the introduction of TMB-5 that reorganizes hydrogen bond within PA66 fiber during process of melting spun, which provides possibility for further optimization of condensed phase structure, resulting in the superior improvement of mechanical performance of PA66.

## 4. Conclusions

Based on the analysis conducted in this study, it can be concluded that the mechanical properties of PA66 fibers can be significantly enhanced through the rational regulation of polymer condensed phase structure. The incorporation of TMB-5 into PA66 fibers facilitates the formation of hydrogen bonds between TMB-5 and PA66 molecular chains and achieve the intermolecular hydrogen bond reorganization within PA66, promoting the movement and alignment of molecular chains during melting spun. This synergistic effect of TMB-5 and the stretching field, results in a substantial increase in the orientation degree of lamellae and the average length of stretched molecular chains within the PA66/TMB-5 fibers. Consequently, PA66/TMB-5 fibers without post-drawing exhibit exceptional tensile strength, reaching up to 226.38 MPa. These findings underscore the importance of understanding and manipulating polymer condensed phase structure for enhancing the mechanical properties of polymers, opening up new avenues for the development of high-performance materials in various industrial applications.

## Data Availability

The original contributions presented in this study are included in the article/Supplementary Material. Further inquiries can be directed to the corresponding author(s).

## Supplementary Materials

The following supporting information can be downloaded at: https://www.mdpi.com/article/10.3390/molecules30040862/s1, Figure S1: Molecular structure of TMB-5; Figure S2: The DSC endothermic curves of TMB-5/PA66 sheets. Figure S3: The loss factor (tanδ) from DMA curves for TMB-5/PA66 sheets. Figure S4: The mechanical properties of TMB-5/PA66 sheets. Table S1: DSC melting data of TMB-5/PA66 fiber. Table S2: The Length of stretched molecules and long period obtained from 2DSAXS results. Table S3: The fiber orientation factor measured by sound velocity method.
