# Supplementary material for: Tailoring Polyamide66 Mechanical Performance: A Strategy for Condensed Phase Structure Optimization Through Hydrogen Bond Reorganization
Source: Molecules. 2025 Feb 13;30(4):862. doi: 10.3390/molecules30040862 (PMC11858307; doi:10.3390/molecules30040862)
Supplement: Supplementary file 1 [file molecules-30-00862-s001.zip › molecules-3421525-supplementary.pdf]

Supporting information

Tailoring PA66 Mechanical Performance: A Strategy for Condensed Phase Structure Optimization through Hydrogen Bond Reorganization

Wen-Yan Wang [1], Pan He [2], Ting Peng [1], Shuai Zhang [1], Guang-Zhao Li [1], Min Nie [3,] and Rui Han [1,*]*

[1] *School of Materials Science and Engineering, Key Laboratory of Materials and Surface Technology (Ministry of Education), Engineering Research Center of Intelligent Air-Ground Integration Vehicle and Control, Xihua University, Chengdu 610039, China*

[2] *Sichuan Provincial Engineering Research Center of Functional Development and Application of High-Performance Special Textile Materials, Chengdu Textile College, Chengdu 611731, China*

[3] *State Key Laboratory of Polymer Materials Engineering, Polymer Research Institute of Sichuan University, Chengdu 610065, China*

* *Correspondence: minnie@scu.edu.cn (M.N.); ruihan_harry@mail.xhu.edu.cn (R.H.)*

1. Supplementary data figure

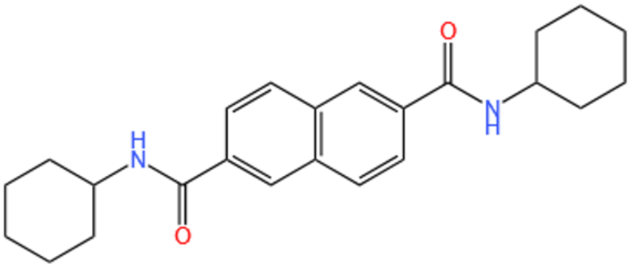

Figure S1. Molecular structure of TMB-5.

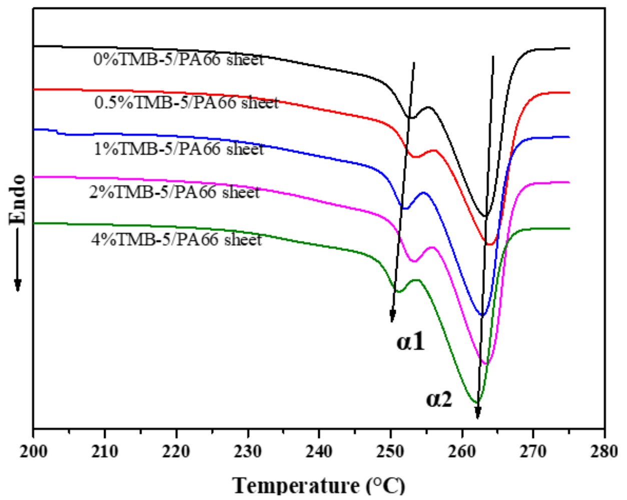

Figure S2. The DSC endothermic curves of TMB-5/PA66 sheets.

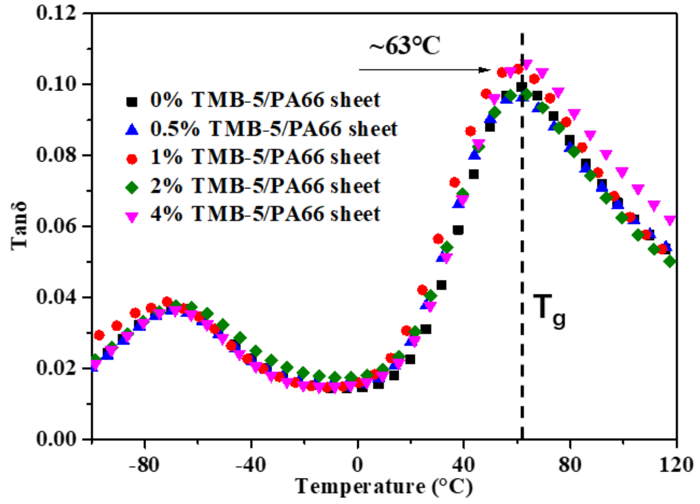

Figure S3. The loss factor (tanδ) from DMA curves for TMB-5/PA66 sheets.

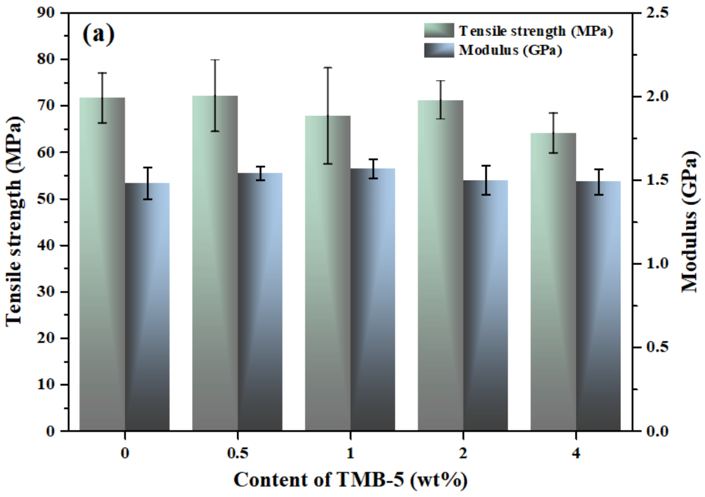

Figure S4. The mechanical properties of TMB-5/PA66 sheets.

2. Supplementary method

Supplementary method S1: The calculation of the orientation degree of lamellae within PA66

As for the orientation degree of lamellae within PA66, utilizing the (100) crystal plane as an indicator, Herman's Orientation Factor was employed for a quantitative comparison of the orientation degree of lamellae within PA66 [1-3]:

$$f = \frac{3\langle cos^2\varphi\rangle - 1}{2} \tag{S1}$$

Therein, $\langle cos^2\varphi\rangle$ is designed as:

$$\langle \cos^2\varphi\rangle = \frac{\int_0^{\frac{\pi}{2}} \mathrm{I}(\varphi)\sin\varphi\cos^2\varphi\,\mathrm{d}\varphi}{\int_0^{\frac{\pi}{2}} \mathrm{I}(\varphi)\sin\varphi\,\mathrm{d}\varphi} \tag{S2}$$

In the equation, φ represents the angle of the given crystal plane normal relative to the reference direction, and I(φ) is the diffraction intensity at the angle φ.

Supplementary method S2: The fitting calculation of average lengths of stretched molecular chains

As for the fitting calculation of average lengths of stretched molecular chains, the Ruland method was employed to analyze the equatorial scattering patterns in the 2D-SAXS graphs [4-6]. Specifically, the nominal azimuthal width (B_{obs}) is a function of $\langle L_{shish}\rangle$ and azimuthal width ($B_{\emptyset}$). The relationship between Bobs and $\langle L_{shish}\rangle$ can be expressed if the Lorentz fit appropriately describes the scattering intensity distribution at a specific q:

$$B_{obs} = \frac{2\pi}{\langle L_{shish}\rangle q} + B_{\emptyset} \tag{S3}$$

Supplementary method S3: The long-period values for the samples

Utilizing equation S4, the corresponding long-period values for the samples can be obtained, which are equivalent to the sum of the thicknesses of individual lamellae and the adjacent amorphous regions [7-9]:

$$L = \frac{2\pi}{q_{max}} \tag{S4}$$

In Equation S4, L represents the long period, and q_{max} is obtained from Figure 4f. Furthermore, by performing one-dimensional integration and Lorentz correction on the results of 2D small-angle scattering, relative q-value curves are obtained (as shown in Fig. 4f), with the peak corresponding to a q value referred to as q_{max}.

3. Supplementary table

Table S1. DSC melting data of TMB-5/PA66 fiber

Samples	Peak temperature (°C)	Melting enthalpy (J/g)	Width of half peak (°C)
PT-0	264.08	71.67	8.70
PT-0.5	263.22	75.51	8.58
PT-1	263.89	75.0	7.94
PT-2	263.67	75.33	8.20
PT-4	262.24	78.67	8.43

Table S2. The Length of stretched molecules and long period obtained from 2D-SAXS results

	0%TMB-5/PA66	0.5%TMB-5/PA66	1%TMB-5/PA66	2%TMB-5/PA66	4%TMB-5/PA66
Length of stretched molecules (nm)	400	454	588	625	671
Long period (nm)	5.51	5.42	5.19	5.46	/

Table S3. The fiber orientation factor measured by sound velocity method

Samples	0%TMB-5/PA66	0.5%TMB-5/PA66	1%TMB-5/PA66	2%TMB-5/PA66	4%TMB-5/PA66
Orientation factor	0.329	0.35	0.386	0.402	0.502

Reference

1. Kong, W.; Li, R.; Zhao, X.; Ye, L. Construction of a highly oriented poly (lactic acid)-based block polymer foam and its self-reinforcing mechanism. *ACS Sustain. Chem. Eng.* **2023**, 11(3), 1133-1145.

2. Liu, Y.; Peng, L.; Lin, J.-L.; Zhou, Y.; Wang, D.-J.; Han, C. C.; Huang, X.-B.; Dong, X. The crystallization behavior regulating nature of hydrogen bonds interaction on polyamide 6, 6 by poly (vinyl pyrrolidone). *Chin. J. Polym. Sci.* **2023**, 41(3), 394-404.

3. Yu, J.; Cheng, H.; Wang, Y.; He, C.; Zhou, B.; Liu, C.; Feng, Y. Multiple shearing-induced high alignment in polyethylene/graphene films for enhancing thermal conductivity and solar-thermal conversion performance. *Chem. Eng. J.* **2024**, 480, 148062.

4. Chen, L.; Xing, C.; Gao, J.; Li, Y.; Wang, Z. Structural evolution of ultra-high molecular weight polyethylene gel film stretching at different temperatures with reservation of shish crystals. *Polymer* **2023**, 284, 126283.

5. Zhang, J.; Usman, K. A. S.; Judicpa, M. A. N.; Hegh, D.; Lynch, P. A.; Razal, J. M. Applications of X-Ray-Based Characterization in MXene Research. *Small Methods* **2023**, 7(8), 2201527.

6. Kim, J.; Heo, S. J.; Lee, D.; Im, B. W.; Kim, T.-H.; Kim, S. G.; Ku, B.-C. Highly strong carbon fibers through synergistic carbonization process of sulfonated poly (p-phenylene

sulfide) and carbon nanotube. *Carbon* **2024**, 219, 118814.

7. Righetti, M. C.; Di Lorenzo, M. L.; Cavallo, D.; Müller, A. J.; Gazzano, M. Structural evolution of poly (butylene succinate) crystals on heating with the formation of a dual lamellar population, as monitored by temperature-dependent WAXS/SAXS analysis. *Polymer* **2023**, 268, 125711.

8. Wang, Z.; Peng, Z.; Balar, N.; Ade, H. Suppressing pre-aggregation to increase polymer solar cell ink shelf life. *J. Mater. Chem. A* **2024**, 12(1), 113-120.

9. Lv, C.; Guo, H.; Yang, E.; Xu, C.; Yan, Q.; Meng, L.; Li, L.; Cui, K. Multiscale Relaxation Behavior of Amorphous Plasticized Poly (vinyl butyral). *Macromol. Rapid Commun.* **2023**, 44(18), 2300226.
